# Severity of developmental delay and parenting behavior in toddlers with neurodevelopmental disabilities

**DOI:** 10.3389/fpsyg.2023.1306227

**Published:** 2024-01-05

**Authors:** Annalisa Castagna, Niccolò Butti, Laura Cordolcini, Mark S. Innocenti, Rosario Montirosso

**Affiliations:** ^1^0–3 Center for the at-Risk Infant, Scientific Institute, IRCCS Eugenio Medea, Bosisio Parini, Lecco, Italy; ^2^PhD Program in Neural and Cognitive Sciences, Department of Life Sciences, University of Trieste, Trieste, Italy; ^3^Institute for Disability Research, Policy & Practice, Utah State University, Logan, UT, United States

**Keywords:** neurodevelopmental disability, parenting behavior, PICCOLO, responsiveness, teaching, developmental delay, parenting stress

## Abstract

**Introduction:**

The presence of a neurodevelopmental disability (ND) represents an adverse condition for child’s development and parent–child relationship, and it is reasonable to assume that the severity of delay may influence parenting behavior. Previous research, however, did not specifically address this issue.

**Methods:**

This cross-sectional study compared parental behaviors of mothers of toddlers with moderate/severe or mild/borderline developmental delay and mothers of toddlers with typical development, while considering maternal emotional states. A total of 88 dyads with children aged between 12 and 47 months participated in a 10-min video-recorded interaction then coded with the PICCOLO, a validated observation checklist that assesses four dimensions of parenting: affection, responsiveness, encouragement, and teaching. The mothers also fulfilled two standardized questionnaires assessing parental stress and presence of depressive symptoms. MANOVA and MANCOVA models were used to explore between-group differences in specific parenting dimensions, also considering parental stress.

**Results:**

Mothers of toddlers with ND were less responsive than the comparison group, while the presence of a moderate/severe developmental delay specifically affected teaching behaviors. No differences emerged for affection and encouragement behaviors. Importantly, although mothers of toddlers with moderate/severe ND reported higher child-related dysfunctional interaction stress, this did not directly affect parenting behaviors.

**Discussion:**

These findings highlight how the presence of a disability and the severity of developmental delay can affect specific dimensions of parenting (i.e., responsiveness, teaching) and might inform clinical practice and research on early parental interventions.

## 1 Introduction

The term neurodevelopmental disability (ND) refers to a wide variety of clinical diagnosis including infant cerebral palsy, genetic syndromes, metabolic diseases and brain injuries that could be related to congenital defects or early at-risk conditions such as preterm birth (Ismail and Shapiro, 2019; Montirosso et al., 2020). These conditions emerge very early in life and may affect the acquisition of basic developmental skills in several domains, from cognitive to behavioral and emotional areas, in a chronologically appropriate manner (Olusanya et al., 2018). Therefore, the presence of a ND represents an adverse condition for child’s development and can strongly influence the early caregiver-child relationship, placing a strain on parenting. Infants and toddlers with ND are indeed less responsive and less engaged in interactions, have limited ability to regulate emotional-behavioral states, and produce less clear emotional signals and social cues (Lipkin et al., 2020). The presence of a ND may affect different parenting dimensions (Norman and Christiansen, 2013; Phillips et al., 2017; Vilaseca et al., 2020). Firstly, the presence of a ND could affect maternal responsiveness and sensitivity, defined as the parents’ ability to adequately read their child’s signals and respond to them in a contingent and emotionally warm way (Shin et al., 2008; Butti et al., 2018). Notably, sensitive and responsive parenting is a salient factor in promoting child’s socio-emotional, behavioral, and cognitive development, and is associated with better developmental outcomes in children with ND (Hutchon et al., 2019; Jeong et al., 2021). Difficulties in interpreting and responding appropriately to infant signals and communications may also lead parents to feel less competent and to experience a critical emotional burden, such as enhanced levels of stress and depressive symptoms (Craig et al., 2016; Scherer et al., 2019). These difficulties might hinder their abilities to provide emotional support and to be emotionally engaged during dyadic interactions, with negative sequelae for child’s developmental outcomes (Green and Baker, 2011). Moreover, the presence of a ND could interfere with parent’s ability to provide appropriate cognitive stimulation and sustain child’s attention (Phillips et al., 2017). These often overlooked parenting behaviors have long-term impacts on cognitive, linguistic, and socio-emotional development (Innocenti et al., 2013; Totsika et al., 2020).

Given the difficulties that can be found in the parent–child relationship in the context of ND, it is reasonable that the severity level of the developmental delay can affect parenting behavior. However, this issue has been scarcely investigated by previous literature (Vilaseca et al., 2020). Developmental standardized scales (i.e., Bayley scales, Griffith’s mental developmental scales) are the golden methods to assess the degree of developmental delay, providing a global index of psychomotor development in terms of age-appropriate skills shown by the child. This developmental quotient index provides a classification of developmental delay in terms of standard deviation from the age-expected score. Although the severity of a disability may be more accurately described as a continuum and the cut-off scores may vary across different batteries, all instruments identify the progressive levels of delay as borderline, mild, moderate and severe, which thus correspond to increasing developmental problems (Cirelli et al., 2015).

Beyond a potential direct impact of the presence of ND on parenting behaviors, a higher level of severity usually requires more care, management and nursing practices (Sterling et al., 2012), with consequent effects on the emotional state and perceived stress of parents (Thurston et al., 2011; Maridal et al., 2021). Previous research reported that parents of children with ND experience significantly higher levels of stress than parents of children with typical development (Pinquart, 2018; Scherer et al., 2019), and developmental delay was found to be associated with mothers’ depression (Vameghi et al., 2016). The high caregiving burden and the increased parental stress and depressive symptomatology can further exacerbate child’s emotional and behavioral problems, starting a vicious circle (Barroso et al., 2018). Thus, it is important to consider the contribution of emotional maternal states (i.e., parental stress and depressive symptomatology) on parenting behaviors in case of ND.

The assessment of parental behaviors linked to developmental outcomes of children with ND is critical to plan intervention aimed to improve early childhood outcomes (Hutchon et al., 2019). To this aim, the Parenting Interaction with Children: Checklist of Observation Linked to Outcomes (PICCOLO) was developed (Roggman et al., 2013b). The PICCOLO is a practical and easy-to-use tool that allows assessing four dimensions of parenting (i.e., affection, responsiveness, encouragement and teaching) for children aged 4–47 months through observation of short play interactions between parents and their children. The PICCOLO is a psychometrically reliable instrument, which has been translated and validated in different cultural contexts (Roggman et al., 2013a) and countries, such as Spain (Vilaseca et al., 2019b), Turkey (Bayoğlu et al., 2013), Brazil (Schneider, 2018), and Italy (Montirosso and Giusti, 2022; Montirosso et al., 2023). Parenting behaviors assessed with the PICCOLO have been shown to be associated with child’s social, cognitive, and language skills both in typical (Roggman et al., 2013a) and atypical development (Innocenti et al., 2013). The PICCOLO was designed for home and care settings of children with ND, with the aim to identify the parenting profile and to promote structured individualized interventions for families based on parent strengths (Norman and Christiansen, 2013; Alves et al., 2022). The advantage of the PICCOLO is that it measures not only affection (i.e., physical and verbal expression of affection, positive emotions, positive evaluation and positive regard) and responsiveness (i.e., reacting sensitively to a child’s cues and expressions of needs or interests and reacting positively to his/her behavior), but also other parental domains such as encouragement, which considers parents’ support of children’s efforts, exploration, independence, play, choices, creativity, and initiative, and teaching, which includes cognitive stimulation, explanations, conversation, joint attention, and shared play. This an important methodological aspect because, although a number of studies have examined affective behavior and responsiveness in parents of children with ND (Warren and Brady, 2007; Bornstein et al., 2008), there is a paucity of research specifically examining other parenting dimensions. Recent studies adopting the PICCOLO have started to shed light on how these parenting behaviors may be differentially affected by the presence of ND (Vilaseca et al., 2020) and are associated with developmental outcomes (Vilaseca et al., 2019a; Rivero et al., 2023).

To further study parenting behaviors in presence of ND, the current study investigated whether and which parenting behaviors in mothers were affected by the degree of severity of child’s ND. With this main aim, parenting behaviors (affection, responsiveness, encouragement and teaching) assessed through the PICCOLO were compared in mothers of toddlers with moderate/severe ND, with mild/borderline ND, and with typical development (TD). Maternal depressive symptomatology and parenting stress were assessed by means of standardized questionnaires and compared between groups; differences were considered in analyses in order to disentangle the role of maternal emotional states on parenting behaviors. According to previous findings (Blacher et al., 2013), we expected that the presence of ND would overall affect parenting behaviors, thus resulting in lower scores in the PICCOLO domains for the clinical groups compared to the TD control sample. However, we also expected that the use of the PICCOLO would allow identifying specific dimensions of parenting, and particularly teaching, affected by the severity of the developmental delay (Vilaseca et al., 2020).

## 2 Materials and methods

### 2.1 Participants

Our sample was composed of 88 Italian mother-toddler dyads including an equal number of children with and without ND and with a chronological age between 12 and 47 months. Although the sample size was not *a-priori* determined by a power analysis, it is in line with recent studies that adopted the PICCOLO to compare parenting behavior in children with and without ND (Vilaseca et al., 2020; Rivero et al., 2023). The clinical group (*N* = 44 dyads; age range: 12.5–46.3 months) was composed of toddlers admitted at the Child Neuropsychiatry and Neurorehabilitation Unit of the Scientific Institute IRCCS “*E. Medea*” (Bosisio Parini, Lecco, Italy), recruited at the beginning of their hospitalization. These toddlers had a diagnosis and/or diagnostic hypothesis for ND (e.g., infantile cerebral palsy, genetic syndrome), and a developmental quotient obtained through standardized assessment (i.e., Griffiths scales), when available, less than 85. The clinical sample was subdivided into two groups according to the level of ND severity. Children with a developmental quotient less than 50 and those whose developmental quotient could not be calculated due to the severity of their disability were included in the moderate/severe disability group (*N* = 22; age range: 13.7–45.5 months); children with developmental quotient greater than or equal to 50 composed the mild/borderline disability group (*N* = 22; age range: 12.5–46.3 months). Apart from the presence of a ND, no other inclusion/exclusion criteria were applied to the clinical groups. While this resulted into heterogeneous samples in terms of diagnoses, this variety was considered to be representative of children with ND usually referred to neuropsychiatry clinical units. A resume of the main diagnoses presented by both the groups is reported in Table 1.

**Table 1:** Main diagnosis presented by children of the two clinical groups.

	**Moderate/severe ND**	**Mild/borderline ND**
Genetic defects/syndromes (e.g., Williams Syndrome)	6 (27%)	6 (27%)
Cerebral palsy	3 (14%)	3 (14%)
Congenital epilepsy/encephalopathy	3 (14%)	1 (4%)
Unspecified psychomotor delay	8 (36%)	10 (45%)
Autism spectrum disorder	1 (4%)	1 (4%)
Sensorineural deficits	1 (4%)	1 (4%)

Data are reported as *N* (%). ND, neurodevelopmental disability.

The control group (*N* = 44 dyads; age range: 12.0–46.6 months) was recruited through contacts with pediatricians or nurseries and was composed of toddlers considered as with typical development (TD). Exclusion criteria were: (i) prematurity, (ii) perinatal or postnatal pathology, (iii) to be referred to the pediatrician for any kind of developmental problem.

For all groups, maternal inclusion criteria were: (i) age older than 18 years; (ii) absence of cognitive impairments and manifest psychiatric disorders; (iii) no single-parent family.

For all groups there were the following maternal exclusion criteria: (i) mother age less than 18 years (ii) limited knowledge and mastery of the Italian language, (iii) presence of psychiatric disorder; (iv) single-parent family.

Parents were invited to participate in this research by assuring them that participation would be entirely voluntarily and informed consent was obtained for all parents. All procedures were in accordance with the 1964 Declaration of Helsinki and its later amendments. Ethical approval was obtained from the Ethical Committee of the Scientific Institute IRCCS “*E. Medea*” (protocol 42/18).

### 2.2 Procedure

This study was conducted at the *0–3 Center for the at-Risk Infant* of the Neuropsychiatry and Neurorehabilitation Unit, which provides clinical psychology services to children with ND hospitalized at the Scientific Institute IRCCS “*E. Medea*” and conducts research with a special focus on the early stages of socio-emotional development. Children, hospitalized with their mothers, participate in a daily diagnostic and/or rehabilitation program (e.g., speech therapy, physical therapy, and so on) and are followed by a multi-professional team using a family-centered approach. The study applied a cross-sectional design in order to compare parenting behavior of mothers of children with and without ND. The protocol included video of mother–child interaction, and completion by the mother of a socio-demographic questionnaire and maternal self-reports questionnaires.

#### 2.2.1 Mother–child interaction

Each mother–child dyad was welcomed into a quiet and comfortable room and, after a brief settling-in phase, mothers were asked to participate in a 10-min video-recorded interaction with their child with the following instructions: “Please, interact and play with your child as you typically do” using an established setting of games: an illustrated book, cubes or interlocking games, a rattle, a plush toy and an electronic game (e.g., robot).

#### 2.2.2 Depressive symptomatology and parenting stress self-rated questionnaires

Maternal emotional state was assessed through two standardized questionnaires, the Beck Depression Inventory (BDI) and the Parenting Stress Index (PSI). The BDI – second edition – is a 21-item self-report instrument developed to measure the presence and intensity of depressive symptoms (Beck et al., 1996). Items are rated on a four-point rating scale, and their sum provides a quantitative measures of depressed feelings/behaviors/symptoms. The BDI demonstrated good internal consistency and has concurrent and discriminant validity in clinical and non-clinical samples (Dozois et al., 1998).

The PSI – fourth edition short form – is a self-report tool, composed by 36 items, that helps to identify the level of parenting stress according to the assumption that parental stress is the joint result of subjective characteristics and a series of aspects closely related to parenting (Abidin, 1997; Johnson, 2015). The scores are aggregated into three subscales: (i) Parental Distress (PD), which measures personal stress factors such as conflict with a partner and daily life restrictions; (ii) Parent–Child Dysfunctional Interaction (PCDI), which assesses parents’ perception of the interaction with their children; (iii) Difficult Child (DC), which captures parents’ perception of their child in terms of behavior and demandingness. The subscale scores are summed up into a total score of parenting stress. For all scales, higher scores indicate higher levels of parenting stress.

#### 2.2.3 PICCOLO coding

The Italian version of the PICCOLO was used to assess parenting (Montirosso and Giusti, 2022; Montirosso et al., 2023). The PICCOLO is composed of 29 items which are scored on a 3-point rating scale, from 0 (absent, no behavior observed) to 1 (some brief or minor behavior) to 2 (clearly, strong or frequent behavior) based on a 10-min video recording parent–child interaction. The items are divided into four domains which measure different aspects of parenting:

- Affection (7 items) involves warmth, physical closeness, and positive expressions toward the child (e.g., shows emotional warmth).
- Responsiveness (7 items) includes parental responses to child’s signals, emotions, words, interests, and behaviors (e.g., pays attention to what the child is doing).
- Encouragement (7 items) includes active support for exploration, initiative, curiosity, creativity, and play (e.g., supports child in doing things in autonomy).
- Teaching (8 items) is the domain which refers to sharing play and interaction, cognitive stimulation, exploration, and questions (e.g., labels objects or actions for the child).

For each domain the mean score given to the individual items was computed. Videoclips coded by two psychologists who completed their training when they presented an interrater agreement of 80% or more with the expert coder (R.M.), following the same criteria as the PICCOLO user’s guide (Roggman et al., 2013b). Eight randomly selected videoclips (four for each group) were coded by both the trained psychologists, and the intraclass correlations coefficients indicated a good agreement between the two independent coders (>0.75).

## 3 Statistical analysis

Preliminary one-way ANOVAs and *χ^2^* tests, were used, respectively, with continuous and categorical variables to verify whether the clinical and the control groups were comparable for age (mothers and toddlers), gender (toddlers), maternal education (years) and scores obtained on the BDI and PSI questionnaires. For the latter, the total score as well as the three subscales were compared, in order to disentangle which subscale significantly contributed to parenting stress. Given that almost half of the sample (*N* = 43) did not disclose their family income due to personal reasons, the mode was used as index to compare the three groups. Then, with the aim to check for differences between parents of toddlers with different levels of ND severity and with TD, the scores assigned on the four PICCOLO scales (affection, responsiveness, encouragement, teaching) were entered in a Multivariate ANOVA (MANOVA) with Group (moderate/severe ND, mild/borderline ND, TD) as between-subject factor. The use of a MANOVA model was chosen as it combines multiple dependent variables in order to examine overall differences between groups, controlling at the same time for the inter-correlations among the dependent variables. Significant between-group differences in the maternal emotional states were considered by inserting the PSI PCDI scale as covariate in a Multivariate analysis of covariance (MANCOVA) model. A significant effect of the covariate and/or its interaction with group would thus point to a critical contribution of maternal stress to parenting behavior, while non-significant effects of the PSI PCDI would indicate that the groups differed in parenting behavior independently from between-group differences in maternal emotional states. Univariate results were then explored to assess between-group differences in specific parenting dimensions, by inserting each parenting scale (i.e., affection, responsiveness, encouragement, teaching) into separate ANOVAs and ANCOVAs models, with group as categorical factor and the PSI PCDI as covariate. The Bonferroni correction was used as post-hoc analysis to examine significant results.

For all statistical tests, significance threshold was set at *p* < 0.05. All data were reported with mean and standard error of the mean (SEM). Effect size was reported as partial eta square (*η^2^_p_*), which estimates the proportion of variance accounted for by each variable. The conventional cut-offs for *η^2^_p_* of 0.01, 0.06, and 0.14 for small, medium, and large effect sizes, respectively, were used (Fritz et al., 2012). We performed all analyses using the Statistica software version 8 (Statsoft, Tulsa, OK).

## 4 Results

Table 2 reports demographic information of the recruited sample and the levels of maternal stress and depressive symptoms assessed through standardized questionnaire.

**Table 2:** Demographic characteristics of the study sample and levels of maternal stress and depressive symptoms (*N* = 88 mother-toddler dyads).

		**Clinical group (ND)**		**Control group (TD)**
		**Moderate/severe**	**Mild/borderline**	
*N* (m: f)		22 (14: 8)	22 (10: 12)	44 (25: 19)
Toddler age (months)		27.1 (10.3)	25.2 (9.7)	25.9 (10.9)
	Mean (SD)			
Mother age (years)		35.5 (4.8)	37.9 (5.4)	37.1 (4.2)
	Mean (SD)			
Maternal education (years)				
	Mean (SD)	14.6 (2.8)	15.8 (3.1)	15.9 (2.7)
Family income (euro)				
	Mode	>25,000, <50,000	>25,000, <50,000	>25,000, <50,000
PSI *Total scores*				
	Mean (SD)	79.3 (12.2)	70.2 (13.1)	69.6 (13.1)
*Parental Distress (PD)*				
	Mean (SD)	25.7 (6.8)	24.8 (6.2)	26.1 (6.9)
*Parent–Child Dysfunctional*				
*Interaction (PCDI)*				
	Mean (SD)	26.3 (6.1)	21.1 (6.3)	18.5 (4.7)
*Difficult Child (DC)*				
	Mean (SD)	27.3 (5.0)	24.3 (5.6)	25.1 (6.0)
BDI score		9.7 (5.3)	8.1 (5.9)	7.5 (3.4)
Mean (SD)				

ND, neurodevelopmental disability; TD, typical development; PSI, parenting stress index; BDI, beck depression inventory.

Preliminary analyses revealed no differences for gender, child age, maternal age and education across groups (all *F* < 1.53, all *χ^2^* < 1.48; all *p* > 0.225). The three groups presented with the same mode of family income, which indicated a middle socio-economic status in line with the general Italian income. Also, the three groups did not differ in terms of depressive symptomatology as assessed by the BDI questionnaire (*F*_2,85_ = 1.60; *p* = 0.208; *η^2^_p_* = 0.04). Conversely, a significant group effect emerged for the PSI (*F*_2,85_ = 4.51; *p* = 0.014; *η^2^_p_* = 0.10), with higher levels of stress reported by mothers of toddlers with moderate/severe disability compared to the TD group (*p* = 0.015), while the other comparisons were non-significant (*p* > 0.064). The analyses on the PSI subscales clarified that the three groups showed comparable stress on PD (*F*_2,85_ = 0.26; *p* = 0.775; *η^2^_p_* = 0.01), which assesses personal factor of maternal distress, and on DC (*F*_2,85_ = 1.72; *p* = 0.185; *η^2^_p_* = 0.04), which is related to the perceived child difficulties. Conversely, mothers of toddlers with moderate/severe disability obtained higher scores in PCDI compared to both mothers of toddlers with mild/borderline disability and the TD control group (*F*_2,85_ = 14.74; *p* < 0.001; *η^2^_p_* = 0.26), thus pointing to the difficulties in building a positive and satisficing relationship with the child as the main source of parenting stress in case of a moderate/severe disability.

**Figure 1:**
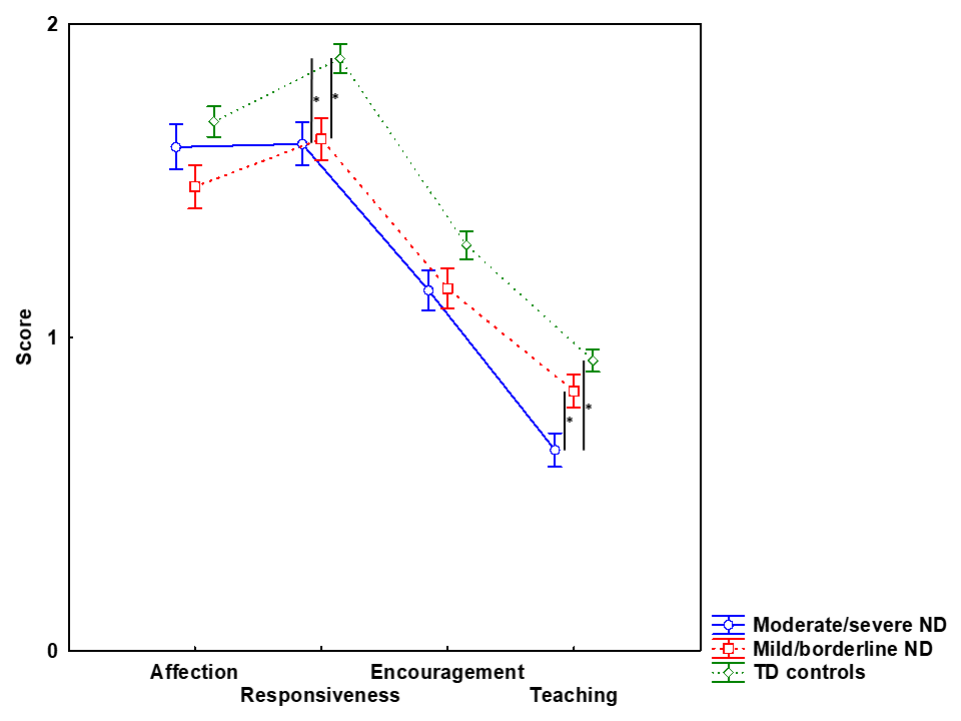
Mean scores of PICCOLO domains for mothers of toddlers with moderate/severe neurodevelopmental disability (ND), with mild/borderline ND and with typical development (TD). Long bar indicates SEM, asterisk represents significant between-groups comparisons at *p* < 0.05.

The MANOVA on the mean scores of the PICCOLO domains yielded a significant effect of group (*F*_8,164_ = 4.50; *p* < 0.001; *η^2^_p_* = 0.18), indicating that overall there were significant between-group differences in parenting behaviors. The following MANCOVA model confirmed that, independently by the maternal stress and specifically the PCDI subscale, the groups showed differences in the PICCOLO scores (*F*_8,162_ = 3.66; *p* < 0.001; *η^2^_p_* = 0.15), further clarified by the univariate results. In detail, the group effect was significant for Responsiveness (*F*_2,84_ = 6.84; *p* = 0.002; *η^2^_p_* = 0.14), showing that the two clinical groups obtained similar scores in this parenting domain (severe/moderate ND: 1.61 ± 0.07, mild/borderline ND: 1.64 ± 0.07; *p* > 0.999), but both were less responsive than TD mothers (1.89 ± 0.05; all *p* < 0.007). A significant between-group difference emerged also for Teaching (*F*_2,84_ = 8.25; *p* < 0.001; *η^2^_p_* = 0.16). Specifically, mothers of toddlers with severe/moderate ND showed fewer teaching behaviors compared to the mild/borderline ND (0.63 ± 0.05 vs. 0.84 ± 0.05; *p* = 0.014) and TD groups (0.93 ± 0.04; *p* < 0.001), while such a difference did not emerge between these two groups (*p* = 0.459). Conversely, no significant between-group differences emerged for Affection (*F*_2,84_ = 2.71; *p* = 0.072; *η^2^_p_* = 0.06) and Encouragement (*F*_2,84_ = 2.30; *p* = 0.101; *η^2^_p_* = 0.05) (Figure 1).

Notably, the covariate PCDI was not significant in either the MANOVA or the univariate models (all *F* < 0.36, all *p* > 0.55), indicating that, even though mothers of toddlers with severe/moderate ND experienced higher levels of dysfunctional interaction-related stress, this did not directly affect parenting behaviors.

## 5 Discussion

The current study investigated the differences in parenting behaviors between mothers of toddlers with ND with different severity levels of developmental delay, and mothers of toddlers with TD. Since higher levels of stress related to parent–child dysfunctional interaction were reported by the group with moderate/severe ND, this subscale of the PSI was included in the analyses. Consistent with the initial hypothesis, between-group differences were detected on specific parenting scales, confirming that the presence and severity of ND affected parenting behaviors.

In accordance with previous literature, both groups with ND were less responsive compared to the control sample, suggesting that, independently by the severity of developmental delay, the presence of a ND may have an impact on parental closeness, parent’s ability to read the child’s cues and to respond contingently. This result confirms responsiveness as one of the main target of early parenting intervention programs for parents of children with NDs (Provenzi et al., 2020; Jeong et al., 2021). On the other hand, as parent–child interaction is a reciprocal process, and as the child’s behavior has an impact on parental behavior, it is important to stress that toddlers with ND, even in presence of mild/borderline developmental delay, provide fewer intelligible cues like subtle changes of emotional expressions and blurred vocalizations, challenging the parent’s ability to understand and expand child’s behaviors (Giusti et al., 2018). In this light, lower levels of responsiveness in mothers of children with ND may result as a response to the demands of parenting a child with lower socio-emotional competence and adaptive behavior.

The severity of developmental delay directly influenced teaching behaviors, with mothers of toddlers with moderate/severe ND obtaining lower scores in this dimension than the other two groups. A similar result was reported in a previous research that adopted the PICCOLO to evaluate parental behaviors in children with ND, with fathers exhibiting less teaching behaviors (Vilaseca et al., 2020). Within a multidimensional framework of parenting of children with ND (Provenzi et al., 2021), these results highlight the importance of considering the specific degree of child’s impairment. Showing fewer teaching behaviors, such as labeling objects or actions and asking the child for information, might represent an implicit modality to provide cognitive stimulation that fits child’s (limited) abilities (Sterling et al., 2012; Blacher et al., 2013). In this vein, showing less teaching behaviors should not be seen as a negative parental attribute *per se*, rather it might be considered as a spontaneous way through which parents of children with severe ND try to adapt to their child’s characteristics. On the other hand, cognitive stimulation, even though apparently too challenging for a child with developmental delay (Engevik et al., 2016), is critical to sustain cognitive development (Malhi et al., 2018), and was found to be a predictor of long-term learning outcomes in at-risk toddlers (Cook et al., 2011). Reminding the Vigotsky’s classical concept of zone of next development (Smagorinsky, 2018), even in presence of a (severe) developmental delay there are assets and potential that can be exploited and expanded within a relational context, and parent–child interaction is the most important context for doing that (Provenzi et al., 2021). As an example, a child may not speak or seem inattentive and unresponsive due to a severe ND; nevertheless, providing verbal cues may help him/her to expand the attentional time window, to create sound-object associations, or simply to direct the gaze toward the mother’s face. In light of these findings, teaching behaviors should be further considered in programming early parenting interventions, in order to help parents provide cognitive stimulation that can foster child’s development.

In keeping with previous literature (Craig et al., 2016; Barroso et al., 2018) mothers of children with moderate/severe ND reported higher levels of stress compared to the other groups, specifically in the subscale assessing the maternal perception of the parent–child relationship (i.e., PCDI). A meta-analysis has documented that this dimension is usually the most affected in case of chronic conditions such as ND (Pinquart, 2018). This subscale taps into the parental perceptions that the child may show few positive behaviors and not much appreciation toward the parent, characteristics that are inherently linked to most kinds of disabilities and not subject to short-term change (Innocenti et al., 1992). Importantly, when inserted as covariate in the models PCDI was non-significant, indicating that parental stress did not directly affect parenting behaviors. However, this result should not lead to underestimate the difficulties and stress experienced by parents of children with ND, also considering the long-term sequelae of prolonged parenting stress on both maternal mental health and child’s developmental outcomes (Azad et al., 2013; Hodes et al., 2017).

The current study confirmed that the PICCOLO can be considered a valid tool to identify the parenting profile with children with different levels of ND. This evidence is important in a clinical perspective as it allows the design of individualized interventions for parents of children with different degrees of developmental delay. For instance, the PICCOLO could be used in combination with Video-Feedback Intervention (VFI), a technique aimed at promoting parent–child interaction in different at-risk and clinical populations (Montirosso et al., 2020; Provenzi et al., 2020). VFI allows parents to observe themselves “from the outside” as they interact with their own child, and it positively impacts caregiving, with benefits for parental sensitivity and interactive attunement (Van Ijzendoorn et al., 2023). The adoption of the PICCOLO could help clinicians to identify strengths and weaknesses of that specific dyad, thus providing individualized targets for the VFI and ultimately facilitating a better fit of parenting behavior to the functioning of the child (Innocenti et al., 2023).

The study has limitations to consider. The sample size was relatively small and included toddlers with different diagnoses, even though they all presented a general developmental delay. The complex nature and variety of the diseases presented by both clinical samples prevented us also from verifying the impact of a specific diagnosis on parenting behavior. As a consequence, caution is warranted in generalizing our finding to specific clinical populations of toddlers with ND (Blacher et al., 2013). This study focused on maternal emotional states and parenting behaviors, but also cognitive factors, such as explicit and implicit parental representations, should be taken into account when assessing and supporting parents of children with ND (Provenzi et al., 2021). Although this work examined the effect of the severity of psychomotor delay on parenting behaviors, a topic that was not directly addressed by previous literature, there are many other child’s characteristics, such as temperament and specific developmental difficulties (e.g., motor or linguistic impairments) that could affect different dimensions of parenting (Sterling et al., 2012). Moreover, this study adopted a cross-sectional design which prevented the evaluation of whether and how parenting behaviors described by the PICCOLO could predict child’s development in conditions of ND. Therefore, longitudinal studies are needed to investigate this critical issue (Montirosso et al., 2020). Lastly, the samples included mothers who all belong to a similar cultural milieu. Future research should verify and extend these results to fathers (Vilaseca et al., 2020) and to families with different cultural backgrounds (Bozicevic et al., 2021).

## 6 Conclusion

This study adopted the PICCOLO to investigate differences in parenting behavior between mothers of toddlers with and without ND. Results highlighted that the presence of ND and the severity of the associated developmental delay can affect specific domains of parenting, specifically responsive and teaching behaviors, regardless of (increased) parenting stress associated with more severe developmental delay. These findings also confirm that the PICCOLO is an easy-to-use, reliable and severity-sensitive instrument, which could be used in clinical practice to assess parental behaviors and design individualized early interventions for parents of children with ND.

## Data availability statement

The dataset used for the analyses in this study is available through a public repository at the following link: https://zenodo.org/record/8402713.

## Ethics statement

Parents were invited to participate in this research by assuring them that participation would be entirely voluntarily and informed consent was obtained for all parents. All procedures were in accordance with the 1964 Declaration of Helsinki and its later amendments. Ethical approval was obtained from the Ethical Committee of the Scientific Institute IRCCS “*E. Medea*” (protocol 42/18).

## Author contributions

AC: Data curation, Investigation, Methodology, Writing – original draft. NB: Data curation, Formal analysis, Investigation, Visualization, Writing – original draft. LC: Data curation, Formal analysis, Methodology, Writing – review & editing. MI: Methodology, Supervision, Writing – review & editing. RM: Conceptualization, Data curation, Formal analysis, Funding acquisition, Methodology, Project administration, Writing – review & editing.
